# Melanoma Clues Beyond Dermoscopic Patterns: Lesion Orientation to Langer’s Lines as a Predictor on the Trunk

**DOI:** 10.3390/cancers17183064

**Published:** 2025-09-19

**Authors:** Umberto Santaniello, Francesco Cavallo, Sara Diana, Silvia Giordano, Orsola Crespi, François Rosset, Andrea Agostini, Giovenale Moirano, Paolo Fava, Pietro Quaglino, Simone Ribero, Paolo Broganelli

**Affiliations:** 1 Section of Dermatology, Department of Medical Sciences, University of Turin, 10126 Turin, Italy; 2 Dermatology and Venereology, Ordine Mauriziano “Umberto I” Hospital, 10128 Turin, Italy; 3 Department of Medical Sciences, University of Turin and CPO-Piemonte, 10121 Turin, Italy

**Keywords:** melanoma, dermoscopy, skin neoplasms, nevi, trunk, regression, Langer’s lines, diagnostic accuracy

## Abstract

Background/Objectives: The diagnosis of melanocytic lesions on the trunk is challenging due to a high frequency of atypical features in benign nevi, leading to a high rate of unnecessary excisions. This study aimed to identify robust dermoscopic predictors of cutaneous melanoma on the trunk and to evaluate a novel diagnostic criterion: the orientation of lesions relative to Langer’s skin tension lines. Methods: We conducted a retrospective analysis of 321 melanocytic lesions (227 nevi and 94 melanomas) excised from the trunk. Dermoscopic features were systematically evaluated. A chi-square test and an age- and sex-adjusted multivariate logistic regression were performed to calculate odds ratios (OR) and identify independent predictors of malignancy. A subgroup analysis was also conducted on “critical” versus “non-critical” anatomical sites. Results: Non-adherence to Langer’s lines was the most powerful predictor of melanoma (OR 5.55, 95% CI 3.22–9.81; *p* < 0.001). Other significant predictors included blue-white veil (OR 5.09) and polymorphous vessels (OR 4.06). Notably, 70% of melanomas did not align with Langer’s lines, whereas 72% of nevi did. Classic features such as scar-like regression were not statistically significant predictors in this cohort. In the subgroup analysis, color asymmetry was a significant predictor of melanoma only in non-critical sites (p for interaction = 0.026). Conclusions: The orientation of a melanocytic lesion relative to Langer’s lines is a powerful and independent predictor of melanoma on the trunk. This simple morphological feature, which may reflect differences in growth patterns between malignant and benign lesions, could serve as an additional clinical cue to support decision-making and improve diagnostic accuracy in this challenging anatomical location.

## Simple Summary

The diagnosis of melanoma on the trunk represents a significant clinical challenge. Benign nevi in this location are subject to mechanical stress, which can induce atypical features and lead to a high rate of precautionary excisions. This study aimed to identify more reliable diagnostic signs for this specific body area. We analyzed 321 melanocytic lesions (227 nevi and 94 melanomas) on the trunk, evaluating several dermoscopic features. We specifically investigated a new concept: whether a lesion’s growth direction relative to the skin’s natural tension lines could distinguish benign from malignant lesions. Our findings show that benign nevi tend to align with these lines, while melanomas grow in a disorganized manner. This simple visual feature may be a powerful predictor of melanoma. This discovery could provide clinicians with a valuable new tool to improve diagnostic accuracy, helping to detect melanomas earlier and reduce unnecessary surgical procedures on the trunk.

## 1. Introduction

**Figure 1:**
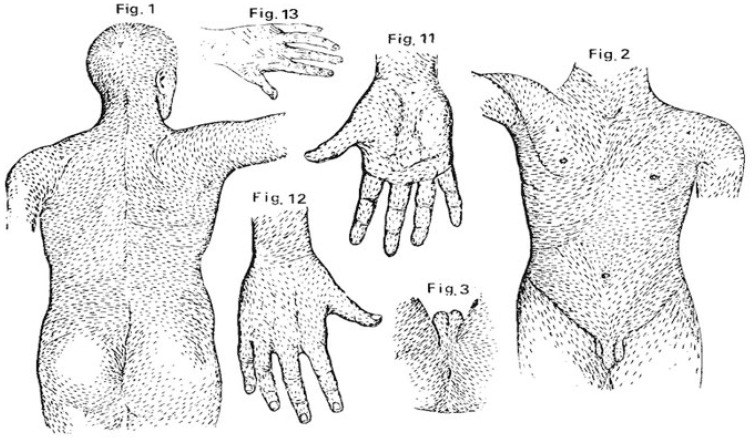
The original map of skin tension lines (cleavage lines) as illustrated by Karl Langer. The image is from Langer’s 1861 publication and is in the public domain. (Source: Langer, K. (1861). Zur Anatomie und Physiologie der Haut. I. Über die Spaltbarkeit der Cutis. Sitzungsberichte der Kaiserlichen Akademie der Wissenschaften, Mathematisch-Naturwissenschaftliche Classe, 44, 19.) [15].

Cutaneous melanoma (CM) represents a significant global health burden, with rising incidence globally. While it represents a minority of skin cancer cases, it is responsible for the majority of skin cancer-related deaths [1]. The prognosis of CM is excellent when diagnosed at an early stage, with 5-year survival rates >99% for localized disease, but drops sharply once metastasis occurs, highlighting the importance of a timely diagnosis [2]. Over the past three decades, dermoscopy has become the standard of care for the evaluation of skin lesions, significantly improving diagnostic accuracy [3,4]. The implementation of systematic algorithms has further refined the diagnostic process [5]. Despite these advances, the diagnostic specificity for melanoma remains suboptimal. This is reflected in the NNE—the number of benign lesions surgically removed to diagnose one melanoma—which can range from 2.2 to over 280 in general practice, indicating a substantial overtreatment [6,7]. A key challenge in dermoscopy is that the morphology of melanocytic lesions is highly dependent on their anatomical location. Well-established “special sites,” such as the face, palms and soles, and nails, exhibit unique dermoscopic patterns with site-specific diagnostic criteria [8,9]. The trunk is the most common location for melanoma, particularly in fair-skinned populations, and it presents its own distinct diagnostic challenges [10]. Mechanical stress and friction can induce atypical features in otherwise benign nevi, mimicking CM, while age further modulates risk, with the trunk representing a frequent site in younger patients [11,12,13]. This ambiguity may contribute to a high rate of excisions and diagnostic ambiguity. An underexplored aspect is the influence of skin biomechanics on lesion morphology. Langer’s lines (Figure 1), which map the direction of minimal skin tension, are fundamental to surgical practice for optimizing cosmetic outcomes but are seldom considered in a diagnostic context [14,15]. A pivotal observation suggested that benign acquired nevi on the back tend to align their major axis with Langer’s lines, presumably due to their slow, indolent growth being influenced by the surrounding dermal collagen architecture [16]. By contrast, we hypothesized that the rapid and disorganized proliferation of CM would not be constrained by these tension lines, causing the lesion to grow in a random orientation. Therefore, the primary aim of this study was twofold: first, to conduct a comprehensive analysis of dermoscopic features of melanocytic lesions on the trunk to identify robust predictors of malignancy in this specific location, and second, to evaluate the orientation of lesions relative to Langer’s lines, considered here as a novel clinical parameter with potential diagnostic relevance.

## 2. Materials and Methods

This study was conducted through a retrospective analysis of clinical and dermoscopic images obtained from a database of skin lesions excised from patients who attended the videodermatoscopy service at the Dermatologic Clinic of the Turin University Hospital, Italy, between 2021 and 2024. The initial database comprised nearly 2100 excised lesions, for which a histopathological diagnosis was obtained. After excluding atypical Spitz tumors due to their uncertain biological nature and diagnostic ambiguity, and cases with missing histopathological reports, incomplete or low-quality clinical or dermoscopic imaging, or incorrect patient data, a total of 338 excisions on the trunk due to clinical suspicion of CM were identified. From this cohort, we selected all melanocytic nevi and CM located on the trunk. The final study population consisted of 227 melanocytic nevi and 94 CM. Clinical and dermoscopic images were acquired using two digital dermoscopy systems: Fotofinder Medicam 1000 (FotoFinder Systems GmbH, Bad Birnbach, Germany) and Vidix 4.0 (Canfield Scientific Inc., Parsippany, NJ, USA). Images were captured in polarized light mode at 10× magnification. All patient data were retrieved from the hospital’s records and subsequently stored in an internal computerized database. For a more detailed topographical analysis, the trunk was subdivided into nine distinct areas: pectoral, sternal/parasternal, abdominal, flank, shoulder, scapular, interscapular, dorsal, and lumbar regions. The Number Needed to Excise (NNE) was defined as the ratio between the total number of lesions excised on suspicion of CM and the number of histologically confirmed melanomas. NNE was calculated separately for lesions located on the trunk and for all other anatomical sites (excluding nails, scalp, and mucosal surfaces). For the study analysis, we gathered histopathological data from the pathology reports, including Breslow thickness and the maximum recorded horizontal dimensions. Each lesion was evaluated for a set of predefined dermoscopic parameters based on the established literature [17,18]. All variables were assessed dichotomously (present or absent), except for the number of colors, which was recorded as a quantitative measure. Each lesion was independently assessed by two dermoscopists with ≥4 years of experience, with arbitration by a senior expert in uncertain cases. Lesion borders were categorized as either faded or sharp, while asymmetry was assessed in terms of both pattern and color. Color evaluation included the total number of distinct colors, as well as the presence of gray. Dermoscopic structures were systematically examined for atypical blotches, hypopigmented structureless areas, atypical network, blue-white veil, scar-like regression, peppering, asymmetric or irregular globules and dots, pseudopods and radial streaks, polymorphous vessels, shiny white structures (white lines), angulated lines, prominent skin markings, and negative (inverse) network. The global dermoscopic pattern of each lesion was further classified as globular, reticular, homogeneous, multicomponent, or non-specific, the latter including featureless, structureless, or feature-poor lesions. When available, digital monitoring data were incorporated to document atypical or asymmetric growth. Lesion orientation was determined by evaluating the alignment of the lesion’s major axis relative to Langer’s lines, which represent the predominant skin tension lines of the anatomical site. For round or symmetrical lesions in which no preferential axis could be established, orientation was conventionally considered parallel to Langer’s lines to ensure consistency of classification. Data were compiled into a dedicated database for statistical analysis. A chi-square (χ^2^) test was used to identify statistically significant differences (*p*-value < 0.05) between nevi and CM for the analyzed variables. Subsequently, a multivariate logistic regression, adjusted for age and sex, was performed for all variables with more than ten occurrences to calculate odds ratios (OR) and their corresponding 95% confidence intervals (CI). A site-specific analysis was also conducted by stratifying the trunk into “critical” areas that were areas subject to greater mechanical stress and tension from upper limb movement (interscapular, scapular, pectoral, and sternal/parasternal) and “non-critical” areas (shoulder, abdomen, dorsal, lumbar, and flank). The *p*-value for interaction was calculated to assess whether the risk associated with each parameter differed significantly between these two subgroups. All analyses were performed using R software (Version 4.4.0 Puppy Cup, released on 24 April 2024).

## 3. Results

### 3.1. Number Needed to Excise (NNE)

A total of 338 excisions were performed in the trunk due to clinical suspicion of CM. Histopathological analysis confirmed 94 melanomas, 227 benign melanocytic nevi, and 17 other non-melanocytic lesions. Based on these data, the NNE for trunk lesions was calculated as 3.6. In comparison, for all other anatomical sites (excluding nails, scalp, and mucosal surfaces), 377 lesions were excised, including 189 CM, corresponding to an NNE of 2.0.

### 3.2. Population Characteristics and Lesion Sites

A total of 321 melanocytic lesions were included in the study (227 nevi; 94 CM). The mean age in the nevus group was 51.3 years (SD 15.1), while the mean age for patients in the melanoma group was 61.5 years (SD 14.7). The mean largest horizontal diameter of lesions was 6.2 mm (SD 2.6 mm) for nevi and 9.6 mm (SD 5.4 mm) for CM. Within the melanoma group, the mean Breslow thickness was 0.4 mm (SD 0.9 mm), with a total of 50 in situ melanomas (53.2%). Regarding patient sex, no significant difference in incidence was noted for CM (52% male, 48% female). In contrast, a higher percentage of excised nevi on the trunk occurred in males (63%) compared to females (37%). The anatomical distribution of lesions is shown in Table 1.

### 3.3. Frequencies of Dermoscopic Features and Adherence to Langer’s Lines in Trunk Nevi and Melanomas

The prevalence of 21 pre-defined dermoscopic features was systematically evaluated in both benign and malignant lesions, and the findings are detailed in Table 2. Several features traditionally considered hallmarks of melanoma were observed at high frequencies in both groups. For instance, an atypical network was present in 79% of melanomas but was also a common finding in 64% of benign nevi. Similarly, structureless areas were identified in 85% of melanomas and 64% of nevi. Irregular globules or dots were identified in 57% of nevi versus only 34% of melanomas. Likewise, radial streaks were present in 32% of nevi compared to 16% of melanomas.

### 3.4. Predictors of Melanoma on the Trunk

To identify independent predictors of malignancy, an age- and sex-adjusted logistic regression analysis was performed (Table 3). After adjustment, features significantly associated with an increased likelihood of melanoma included non-adherence to Langer’s lines (OR 5.55, 95% CI 3.22–9.81; *p* < 0.001), hypopigmented structureless areas (OR 3.05, 95% CI 1.61–6.13; *p* < 0.001), blue-white veil (OR 5.09, 95% CI 2.26 12.04; *p* < 0.001), and polymorphous vessels (OR 4.06, 95% CI 1.67–10.19; *p* = 0.002). Conversely, in our cohort, the analysis identified features predictive of a benign diagnosis. The presence of a globular pattern (OR 0.24, 95% CI 0.07–0.63; *p* = 0.004) and irregular globules or dots (OR 0.46, 95% CI 0.27–0.78; *p* = 0.004) was significantly associated with a benign diagnosis.

### 3.5. Lesion Orientation Relative to Langer’s Lines in CM vs. Nevi

**Figure 2:**
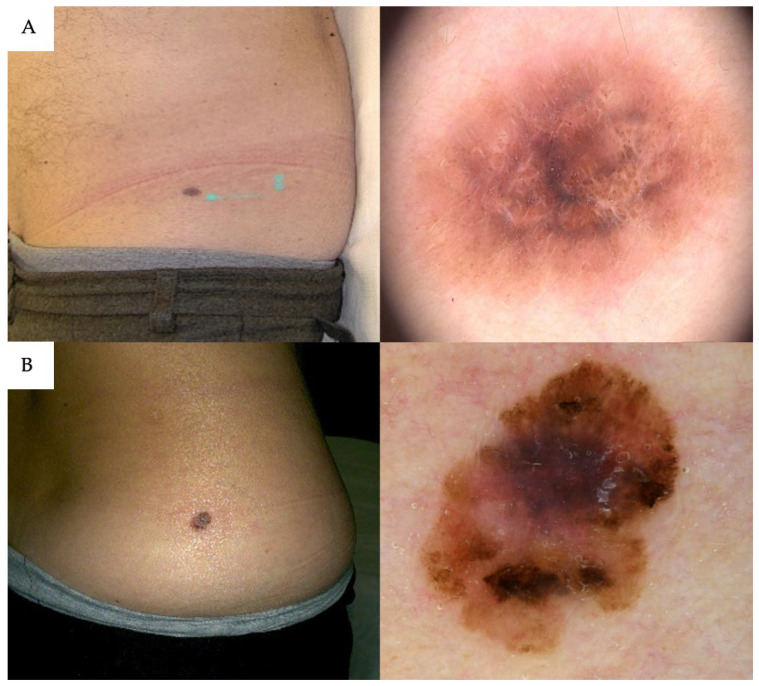
(**A**) A flank nevus with gray color and inverse network at dermoscopy that follow Langer’s lines; (**B**) a CM showing a growth pattern that does not respect skin tension lines (cleavage lines) as illustrated by Karl Langer.

The orientation of the lesion’s main axis relative to Langer’s lines was evaluated. Overall, 72% (163/227) of benign nevi were oriented parallel to Langer’s lines, whereas 70% (66/94) of melanomas were not (Figure 2). A melanocytic lesion on the trunk that was not oriented along Langer’s lines had over five times the odds of being a CM compared to an aligned lesion (OR 5.55, 95% CI 3.22–9.81; *p* < 0.001).

### 3.6. Subgroup Analysis by Anatomic Location: “Critical” vs. “Non-Critical” Sites

A subgroup analysis was conducted by dividing the trunk into “critical” sites (scapular, interscapular, pectoral, sternal/parasternal) and “non-critical” sites (shoulder, abdomen, dorsal, lumbar, flank). Most dermoscopic predictors demonstrated a consistent effect across both subgroups. Non-adherence to Langer’s lines was a significant predictor of melanoma in both critical (OR 5.29, 95% CI 2.43–12.16; *p* < 0.001) and non-critical (OR 6.04, 95% CI 2.72–14.14; *p* < 0.001) areas. Features predictive of nevi, such as the globular pattern and radial streaks, also retained their significant association in both subgroups (Table 4). A statistically significant interaction was found for asymmetry of color (*p* for interaction = 0.026). In non-critical sites, color asymmetry was a significant predictor of melanoma (OR 2.27, 95% CI 1.06–5.02; *p* = 0.035). In critical sites, color asymmetry was not associated with malignancy (OR 0.63, 95% CI 0.29–1.35; *p* = 0.234).

## 4. Discussion

The differential diagnosis of melanocytic lesions on the trunk represents a significant daily challenge for dermatologists, an opinion corroborated by the findings of this study. Our data reveal an NNE of 3.6 for the trunk, a figure markedly higher than the 2.0 for all other anatomical sites combined, confirming that truncal lesions are more frequently excised for histopathological assessment. This low NNE reflects the nature of our database, which includes only lesions referred for second-level evaluation, thus representing a preselected population. However, the relatively higher NNE for truncal lesions, in our cohort, quantifies a site-specific diagnostic uncertainty that persists even within a specialist setting and aligns with the broader clinical challenge reflected in the literature [6,7]. To further characterize this diagnostic uncertainty, we analyzed the prevalence of 21 predefined dermoscopic features in both benign and malignant truncal lesions (Table 2). Several features classically considered hallmarks of melanoma were detected at unexpectedly high frequencies in nevi [4,17,19]. For example, an atypical network was observed in 79% of melanomas but also in 64% of nevi, and hypopigmented structureless areas were present in 85% of melanomas and 64% of nevi. Moreover, dermoscopic features such as irregular globules/dots and radial streaks—observed in melanomas but also in growing or dysplastic nevi—were significantly more prevalent in the nevus cohort (irregular globules/dots detected in 57% of nevi versus 34% of CM; radial streaks observed in 32% of nevi compared to 16% of CM) [20,21]. This striking overlap highlights the reduced specificity of major melanoma criteria in this anatomical site and quantitatively supports the clinical impression that traditional diagnostic algorithms may perform worse on the trunk. These findings reinforce the notion that truncal lesions display a high degree of phenotypic convergence between benign and malignant patterns, increasing the risk of both false-positive and false-negative interpretations. Our hypothesis theorizes that this difficulty arises not from a unique microanatomy, as seen in “special sites”, but from the trunk’s distinct biomechanical stress. The constant tensile and frictional forces may induce morphologic changes in benign nevi that mimic the dermoscopic features of malignancy, acting as a significant confounder. In interpreting our results from this perspective, we first validated our cohort against previous studies. An age- and sex-adjusted logistic regression analysis demonstrated that established predictors of CM, such as the blue-white veil (OR 5.1) and polymorphous vessels (OR 4.1), retained strong predictive power, consistent with a meta-analysis by Williams et al., which reported ORs of 6.3 and 5.1, respectively [20]. However, in our cohort, scar-like regression was not a statistically significant predictor of CM (OR 1.3; *p* = 0.1111), a complete contrast to its recognized role as a hallmark of malignancy [22]. This finding is further supported by the lack of significance for the presence of the gray color, a feature histologically linked to regression [23]. From the perspective of our working hypothesis, this anomaly can be explained: chronic microtrauma on the trunk may induce “pseudo-regression”—inflammation, fibrosis, and melanophages—in benign nevi, creating features that are histologically and dermoscopically indistinguishable from true, immune-mediated regression in melanoma [24,25,26,27]. This high prevalence in the benign control group effectively invalidates the feature’s specificity. The most significant and novel finding of this study, however, provides a new lens through which to view these biomechanical interactions. We identified that the lesion’s major axis orientation relative to Langer’s lines is a powerful predictor of malignancy in melanocytic neoplasms. A lesion whose major axis does not follow these lines of skin tension has over five times the odds of being a CM (OR 5.55; 95% CI 3.22–9.81; *p* < 0.001, adjusted for sex and age). This elevates a previous qualitative observation by McClenahan et al. into a quantified, clinically potent tool [16]. The implications of this finding can be discussed in the broadest context of cell biology and tumor mechanics. Langer’s lines represent the macroscopic manifestation of the dermis’s anisotropic architecture, where collagen fibers are preferentially oriented [28,29]. It is a well-established principle that cell migration is influenced by the topography of the extracellular matrix (ECM), a phenomenon known as “contact guidance” [30]. We propose that the slow, organized proliferation of benign nevus cells is guided by this dermal architecture, causing the lesion to elongate along the path of least mechanical resistance, parallel to Langer’s lines. Malignant melanocytes, conversely, grow in a more chaotic manner. In this state, malignant cells are capable of greater individual motility and active ECM remodeling; therefore, they can grow without adhering to the tissue’s intrinsic architecture, invading in a direction dictated by their own proliferative driver rather than by pre-existing tension lines [31,32]. Therefore, a lesion’s macroscopic orientation becomes a visual proxy for its microscopic biological behavior: alignment signifies adherence to tissue rules (benign), while non-alignment signifies chaotic invasion (malignant). This hypothesis is further corroborated by computational modeling studies. A study by Crisan et al. developed a computational model simulating the growth of melanocytic nevi under mechanical stress [33]. Their model predicted that benign lesions, when subjected to the anisotropic tension characteristic of skin, naturally evolve into an elliptical shape, with their major axis aligning with the direction of principal stress—a finding that provides a compelling theoretical basis for our observation of nevi aligning with Langer’s lines. Furthermore, the model suggested a mechanistic link to malignant progression. As the simulated nevus grows, internal mechanical stress accumulates, particularly at the periphery. The authors proposed that if this stress surpasses a critical threshold, it can induce a loss of cellular cohesion at the border, a physical event that could represent the initial step towards invasion [33]. This concept aligns remarkably well with our finding that CMs disregard these lines of tension, suggesting their growth is driven by an active state that overcomes the passive guidance of the dermal microenvironment. This interplay was further highlighted in our subgroup analysis of “critical” (high-stress) versus “non-critical” (low-stress) truncal sites. While most predictors were consistent, color asymmetry was a significant predictor of CM only in non-critical sites, losing all significance in critical areas (*p* for interaction = 0.026). This suggests that in high-stress regions, chronic mechanical forces can induce benign pigmentary variegation, rendering color asymmetry an unreliable marker. The clinical implications of these findings are substantial. The Langer’s lines criterion, if validated, could be integrated into diagnostic algorithms, potentially improving accuracy and reducing the NNE for the trunk. However, this study has limitations, including a selection bias inherent in using only excised nevi and the retrospective nature of the analysis, which was not optimized for assessing lesion orientation. These limitations highlight clear future research directions. It is crucial to conduct a multicentric, prospective study with a standardized photographic protocol to validate the Langer’s lines criterion. Furthermore, in vitro studies using anisotropic collagen scaffolds could directly test the contact guidance hypothesis by observing the migration patterns of benign versus malignant melanocytes. In conclusion, this study not only provides data confirming the diagnostic complexity of the trunk but also offers a novel, powerful, and biologically plausible criterion—lesion orientation—that could transform clinical practice for this challenging anatomical site, pending further validation.

## 5. Conclusions

Our findings indicate that the trunk is a site of significant diagnostic complexity for melanocytic lesions, as demonstrated by an NNE higher than at other anatomical sites (3.6 vs. 2.0). We found that the diagnostic utility of established CM predictors, particularly regression, is attenuated on the trunk, likely due to biomechanical confounding factors that induce pseudo-atypical features in benign nevi. This finding helps explain the clinical challenge and high rate of benign excisions in this location. The most significant contribution of this work is the identification and quantification of a novel and powerful predictor of malignancy: the non-alignment of a lesion with Langer’s skin tension lines. A melanocytic lesion on the trunk that does not follow this orientation has 5.55 times the odds of being a melanoma. This simple, overlooked feature, supported by a robust biomechanical rationale and consistent with computational models of tumor growth, offers a promising tool to improve diagnostic accuracy. Future multicentric prospective studies are essential to validate this finding, which, if confirmed, could be integrated into diagnostic algorithms to facilitate earlier melanoma detection and reduce unnecessary excisions.

## Figures and Tables

**Table 1:** Anatomical site/area of excised lesions on the trunk.

Site/Area	Nevi, *n* (%)	CM, *n* (%)
Interscapular	39 (17.2%)	14 (14.9%)
Scapular	43 (18.9%)	16 (17.0%)
Pectoral	22 (9.7%)	10 (10.6%)
Sternal/Parasternal	7 (3.1%)	9 (9.6%)
Dorsal	29 (12.8%)	13 (13.8%)
Lumbar	27 (11.9%)	7 (7.4%)
Abdomen	38 (16.7%)	16 (17.0%)
Flank	13 (5.7%)	5 (5.3%)
Shoulder	9 (4.0%)	4 (4.3%)
**Total**	**227 (100%)**	**94 (100%)**

**Table 2:** Frequency of dermoscopic features and adherence to Langer’s lines in truncal nevi versus melanomas.

Parameter	Nevi, *n* (%)	CM, *n* (%)	*p*-Value
Asymmetry of pattern	107 (47%)	60 (64%)	**0.0052**
Asymmetry of color	120 (53%)	58 (62%)	0.1911
Atypical blotches	23 (10%)	21 (22%)	**<0.01**
Blue-white veil	11 (5%)	21 (22%)	**<0.01**
Hypopigmented structureless areas	145 (64%)	80 (85%)	**<0.01**
Atypical network	145 (64%)	74 (79%)	**0.0114**
Regression (scar-like)	34 (15%)	21 (22%)	0.1111
Peppering	48 (21%)	33 (35%)	**<0.01**
Irregular globules/dots	130 (57%)	32 (34%)	**<0.01**
Pseudopods	11 (5%)	1 (1%)	0.1041
Radial streaks	73 (32%)	15 (16%)	**<0.01**
Polymorphous vessels	11 (5%)	14 (15%)	**<0.01**
Shiny white lines	16 (7%)	11 (12%)	0.1279
Angulated lines	5 (2%)	11 (12%)	**<0.01**
Inverse network	14 (6%)	6 (6%)	0.9420
Prominent skin markings	2 (1%)	2 (2%)	0.5956
Gray color	102 (45%)	47 (50%)	0.4497
Globular pattern	52 (23%)	4 (4%)	**<0.01**
Reticular pattern	132 (58%)	42 (45%)	**0.0228**
Homogeneous pattern	20 (9%)	4 (4%)	0.1579
Multicomponent pattern	66 (29%)	46 (49%)	**<0.01**
Non-adherence to Langer’s lines	64 (28%)	66 (70%)	**<0.01**

Statistical analysis performed using the chi-square (*χ*^2^) test. Statistically significant values (*p* < 0.05) are bolded highlighted.

**Table 3:** Age- and sex-adjusted logistic regression analysis of dermoscopic predictors for truncal melanoma.

Parameter	Odds Ratio (OR)	95% Confidence Interval (CI)	*p*-Value
**Predictors of Melanoma (OR > 1)**			
Non-Adherence to Langer’s Lines	5.55	3.22–9.81	**<0.001**
Angulated Lines	5.24	1.56–20.97	0.07
Blue-White Veil	5.09	2.26–12.04	**<0.001**
Polymorphous Vessels	4.06	1.67–10.19	**0.02**
Hypopigmented Structureless Areas	3.05	1.61–6.13	**<0.001**
Atypical Blotches	2.35	1.16–4.76	0.18
Multicomponent Pattern	2.01	1.18–3.42	0.10
Peppering	2.30	1.07–3.37	0.29
Atypical Network	2.24	1.03–3.41	0.40
**Predictors of Nevi (OR < 1)**			
Globular Pattern	0.24	0.07–0.63	**0.04**
Radial Streaks	0.41	0.21–0.77	0.06
Irregular Globules/Dots	0.46	0.27–0.78	**0.04**

Odds ratios are adjusted for patient age and sex. Variables with fewer than 10 cases (e.g., pseudopods, documented growth) were excluded from the regression model. Statistically significant values (*p* < 0.05) are highlighted.

**Table 4:** Subgroup logistic regression analysis of dermoscopic predictors by anatomic site.

Parameter	Non-Critical Sites, OR (95%CI)	Critical Sites OR (95%CI)	*p* for Interaction
Non-Adherence to Langer’s Lines	6.04 (2.72–14.14)	5.29 (2.43–12.16)	0.958
Hypopigmented structureless areas	2.19 (0.96–5.33)	5.15 (1.76–19.16)	0.461
Blue-White Veil	3.67 (1.00–14.72)	6.44 (2.21–20.78)	0.415
Asymmetry of Color	2.27 (1.06–5.02)	0.63 (0.29–1.35)	**0.026**
Atypical Network	2.81 (1.15–7.70)	1.44 (0.65–3.35)	0.240
Multicomponent Pattern	1.35 (0.60–2.97)	2.60 (1.23–5.57)	0.379
Radial Streaks	0.68 (0.29–1.55)	0.16 (0.04–0.48)	0.097
Globular Pattern	0.32 (0.05–1.27)	0.20 (0.03–0.78)	0.944

Odds ratios are adjusted for patient age and sex. Only key variables are shown for brevity. Statistically significant interactions (*p* < 0.05) are highlighted.

## Data Availability

The raw data supporting the conclusions of this article will be made available by the authors on request.

## Abbreviations

The following abbreviations are used in this manuscript:

CM	Cutaneous melanoma
SD	Standard deviation
OR	Odds ratio
NNE	Number needed to excise
ECM	Extracellular matrix
